# Knowledge, attitudes and practices of East Flemish general practitioners towards subscribing LARCs for adolescents

**Published:** 2018-03

**Authors:** I Maes, D Van Braeckel, K Michielsen

**Affiliations:** International Centre for Reproductive Health, department of Uro-Gynaecology, faculty of Medicine and Health Sciences, Ghent University, De Pintelaan 185 UZP114, 9000 Gent, Belgium.

**Keywords:** adolescents, contraceptive use, general practitioners, Long-Active Reversible Contraceptives (LARCs), IUD

## Abstract

**Objective:**

While long acting reversible contraceptives (LARCs) offer a more reliable protection against unintended pregnancies than short acting reversible methods (SARCs), their uptake among adolescents in Flanders (Belgium) is low. This study assesses to what degree general practitioners constitute a barrier for the uptake of LARCs by adolescents.

**Methods:**

We did an online survey among 79 general practitioners in East Flanders to assess their knowledge, attitudes and behaviours related to advising and prescribing LARCs to adolescents.

**Results:**

Almost one third (31,6%) of respondents does not discuss LARCs with adolescents and a vast majority (87.3%) indicates to only recommend SARCs. Uncertainty of their own technical skills is among the main barriers, next to the perceived need to transfer the patient to a gynaecologist. Half of the respondents indicate that their practice is equipped to place implants and hormonal IUDs, one in four to place copper IUDs. Furthermore, responses indicate that prejudices and traditions play a role in the reluctance of general practitioners to recommend LARCs to adolescents.

**Discussion:**

These results indicate that adolescents are not always offered the necessary information to make an informed choice between a full range of modern contraceptives. Another worrying finding is that most of the main reasons for hesitating to recommend LARCs to adolescents are provider-related barriers rather than reasons related to the well-being of the patients.

**Conclusion:**

Based on the data, we can say that (lack of) knowledge, skills and equipment of general practitioners constitute a barrier to uptake of LARCs by adolescents.

## Introduction

According to a large-scale population survey conducted in 2011-2012, one out of four pregnancies in Flanders is unplanned and almost one out of five was initially unwanted.(Buysse et al. 2013) Even if teenage deliveries are rather rare in Flanders, unintended pregnancies occur, as is indicated by the relatively high number of abortions in the adolescent age group: in 2011, 13.6% (2,662) of all abortions in Belgium were performed on girls and women aged 10-19, and another 25.7% (5,027) involved women of 20-24 years old (Nationale Commissie voor de Evaluatie van de Wet van 3 april 1990 betreffende de zwangerschapsafbreking 2012). This points at non-use or sub-optimal use of contraception by young people.

Contraceptive use among young people is rising in Flanders, Belgium and even doubled between 2005 and 2015. In 2015 58% of all young people aged 15-21 years reported using a reliable contraceptive method. The contraceptive pill is the most commonly used method: 91% of young people using contraception is using the pill. Nine percent is using another method, mainly the implant, the patch, the vaginal ring and the intra-uterine device (IUD). (Algemene Pharmaceutische Bond, 2016)

Long acting reversible contraceptives (LARCs) as the intra-uterine devices (IUD) and implants offer a more reliable protection against unintended pregnancies than short acting reversible methods (SARCs). Under typical use, the percentage of women experiencing a pregnancy during the first year of use is estimated at 0.8%, 0.2% and 0.05% for cupper IUD, hormonal IUD and implant respectively, compared to for instance 18% for male condom and 9% for combination pill (World Health Organization, 2016). Furthermore, LARCs are more cost-effective than short-action reversible contraceptives in the long run.

Given the fact that LARCs offer a considerably better protection against unplanned pregnancy than other reversible methods, one would expect a relatively high use of this contraceptive method, also among adolescents. However, while use of LARCs has substantially risen, it remains rare compared to its use among older women. (Algemene Pharmaceutische Bond, 2016) After new refunding rules in 2013 made the IUD free for young people up to 21 years its number of users increased from about 3.850 users in 2013 to 10.360 (2,8%) in 2015 compared to 213.600 or 10,3% of women aged 22-49 years. (Algemene Pharmaceutische Bond, 2016) The use of the implant also increased, but more slowly from 3.986 in 2013 to 5.306 (1,4%) in 2015 compared to 15.700 women (0,7%) aged 22-24 years in 2015.

The choice for a contraceptive method is influenced by a large number of factors. Hence, several reasons can be considered for the underuse of LARCs, an obvious one being a lack of accurate information: LARCs are relatively unknown among women, and at the same time many women overestimate the effectiveness of condoms and pills.(Branum and Jones, 2015) One of the possible causes of this information gap may be found in inadequate counselling by general practitioners. Most contraceptives can only be obtained on subscription, so girls have to see either a general practitioner or a gynaecologist, who then has the opportunity to inform the patient about all possibilities and to discuss which option(s) would be most appropriate for the patient.

This study aims to assess to what degree general practitioners constitute a barrier for the uptake of LARCs by adolescents.

## Materials and methods

This is a quantitative study with a cross-sectional study design that uses an online survey (LimeSurvey) to collect data. After a preliminary literature search, an online survey was developed. This survey consisted of five parts (25 questions): socio-demographic factors, prescription behaviour, knowledge related to LARCs, and cases. The survey was pre-tested among two general practitioners.

The survey was sent by email to all general practitioners in East Flanders through mediation of the ‘Orde der Artsen’ of East Flanders (professional federation of physicians). In addition, the survey was sent by e-mail to all coordinators of LOK (local quality groups of physicians) in East Flanders, with the request to forward it to their members. Furthermore, the link to the survey was sent to e-mail addresses of general practitioners in East Flanders that could be found on the Internet.

Ethical clearance was obtained from the Ethical Committee of Ghent University Hospital, and all respondents had to give their informed consent electronically before being able to access the questionnaire.

The data were exported from LimeSurvey to SPSS. Correlations between variables and differences between groups were tested with Chi-squared test, t-tests and Wilcoxon Sign Rank test.

## Results

### Socio-demographic characteristics

In total, the sample included 79 respondents, which corresponds with a response rate of 4,2%. The average age of the respondents was 43 years (median=35) and 54.4% were women. Women are overrepresented in the youngest age groups and underrepresented in the older ones, which corresponds with the distribution of the overall population of general practitioners in Flanders. (Federale Overheidsdienst Volksgezondheid 2016) 72,2 % of the respondents works in a group practice, with an overrepresentation of young practitioners and women (TableI).

**Table I:** Socio-demographic characteristics of respondents.

	Total	Female	Male	p-value
Group practice (Yes) - N(%)	57 (72,2%)	37 (86%)	20 (55,6%)	P=0,003
Age (mean(sd)/median/min-max)	43(sd 15)/35/26-73	36(sd 10)/31/26-63	52(sd 15)/57/27-73	P<0,001
Total	79 (100%)	43 (54,4%)	36 (45,6%)	

### Discussing and recommending LARCs

Asked which contraceptives are most often recommended for adolescents (more than one contraceptive method could be indicated), 96.2% refers to the combination pill. Ten percent says to often recommend the hormonal IUD. The other LARCs scored lower (5.1% for the cupper IUD and 3.8% for the implant). Questionning which methods are least or not recommended for adolescents, the implant received the highest score with 75.9%, followed by the cupper IUD (68.4%) and the hormonal IUD (59.5%). When an adolescent patient has already made her choice before the consult, 13.9% of the respondents does not counsel and discuss any other method, while about half of the respondents mentions the hormonal IUD (less for the copper spiral and the implant). Almost one third (31,6%) of the respondents does not counsel and discuss LARCs with adolescents, and a vast majority (87.3%) indicates recommending only SARCs (Table II).

**Table II:** Contraceptive methods that are most/least recommended to adolescent patients and contraceptive method that respondents inform patients about, even if the adolescent patient has already made a contraceptive choice.

Contraceptive method	Most recommended* %	Least recommended* %	Most discussed* %
Combination pill	96,2	2,5	77,2
Depo-Provera	13,9**	50,5	32,9
Progestin-only pill	6,3	55,7	19
Contraceptive patch 1,3 55,9	1,3	55,9	19
Vaginal ring	16,5	13,9	58,2
Copper IUD	5,1	68,4	21,5
Hormonal IUD	10,1**	38***	49,4
Implant	3,8	75,9	22,8
I do not talk about other methods			13,9
Only SARCs	87,3		31,6
Only LARCS	2,5		1,3
Both	10,1		53,1

*Respondents could choose up to three methods **Significantly more recommended by female doctors than by male doctors ***Significantly less recommended by male doctors than by female doctors

The respondents were asked to indicate – per LARC – the main reasons why they would not recommend LARCs to adolescents. Within LARCs, we see that the uncertainty of their own technical skills is among the main barriers. Other important barriers are the perceived need to transfer the patient to a gynaecologist and the fact that placing and removing the contraceptive depend on intervention from a doctor. For the copper IUD an important barrier are the side effects (Table III)

**Table III:** Main reasons not to recommend LARCs.

	Implant	Hormonal IUD	Copper IUD
The necessity to refer to a gynaecologist	30,6% (N=22)	43,1% (N=31)	44,4% (N=32)
Uncertainty about one’s technical skills	40,3% (N=29)	51,4% (N=37)	47,2% (N=34)
Duration of the contraceptive method	37,5% (N=27)	27,8% (N=20)	19,4% (N=14)
Pain threshold of the patient	12,5% (N=9)	29,2% (N=21)	27,8% (N=20)
Insertion and removal depend on intervention of a medical doctor	48,6% (N=35)	36,1% (N=26	36,1% (N=26)
Cost	26,4% (N=19)	31,9% (N=23)	5,6% (N=4)
Side effects (e.g. blood loss)	30,1% (N=22)	23,3% (N=17)	50,7% (N=37)

### Knowledge and skills

Respondents were asked to self-assess their knowledge and skills related to LARCs. For the IUDs, a clear majority considers his/her knowledge as ‘very good’ or ‘fair’, though the hormonal IUD is significantly better known than the cupper IUD, for which almost 1 in 5 respondents consider their knowledge as ‘limited’. For the implant, 30.1% of respondents indicate that their knowledge is ‘limited’ (Table IV).

**Table IV:** Self-perceived knowledge of respondents regarding LARCs (n=73).

	Very good	Fair	Limited
Implant	19 (26%)	32 (43,8%)	22 (30,1%)
Hormonal IUD	38 (52,1%)	30 (41,1%)	5 (6,8%)
Copper IUD	32 (43,8%)	28 (38,4)%	13 (17,8%)

**Figure 1:**
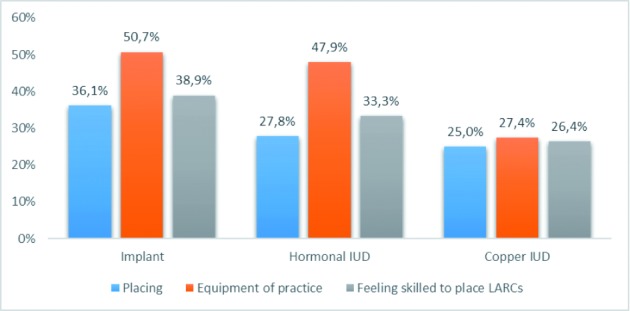
— Placing LARCs, equipment of practice, feeling skilled to place LARCs.

One respondent out of four inserts IUDs him- or herself. For implants this is 36,1%. 38,9% says to feel sufficiently skilled to place an implant. For the hormonal and copper IUD this is less; respectively 33.3% and 26.4%. Half of the respondents indicate that their practice is equipped to place implants and hormonal IUDs, one in four to place copper IUDs. (Figure 1).

### Propositions

The respondents were asked to evaluate two propositions with ‘right’ or ‘wrong’. These propositions were based on prejudices that were mentioned in the literature.

‘Long acting reversible contraceptives are more expensive than short acting reversible contraceptives on the long term.’ 14.3% of the respondents agrees with this untrue statement. Age and gender seem to play an important role here: the average age of the respondents who agreed with the statement was 62.5 years, compared to 34 years for the respondents who disagreed, and also significantly more female doctors (30 vs 17) correctly answered the statement.

‘The risk of an unintended pregnancy is in practice higher among patients that use the combination pill than among patients that use a copper IUD.’ Despite the considerable difference in Pearl index in favour of hormonal IUD, 31.4% of respondents does not agree with the proposition. Also here, more female (22 vs 14) and more younger doctors answered the statement correctly, though for the former the difference was not statistically significant.

### Cases

The last part of the survey consisted off three cases. Respondents were requested to indicate for each of these cases which contraceptive method(s) they would recommend.

*Case 1*: ‘An 18-year old girl with a boyfriend would like to start contraception . What would you advise her?’ Only 22% would recommend the hormonal IUD, and the other LARCs received even lower scores (Table V).

**Table V:** Recommended contraceptive method in three cases

	Case 1	Case 2	Case 3
Condom*	57,4 %		
Combination pill	94,1 %	82,4 %	29,4 %
Depo-provera	16,2 %	5,9 %	51,5 %
Progestin-only pill	11,8 %	19,1 %	2,9 %
Vaginal ring	50 %	52,9 %	52,9 %
Contraception patch	11,8 %	13,2 %	20,6 %
Implant	5,9 %	16,2 %	26,5 %
Hormonal IUD	22,1 %	55,9 %	57,4 %
Copper IUD	11,8 %	27,9 %	20,6 %

*In cases 2 and 3 ‘Condom’ was not an answer option.

*Case 2*: ‘A 22-year old women with one child would like to postpone a second pregnancy. In the meantime she would like to use a contraceptive method that she can stop when she would be ready for a second pregnancy. She thinks this might be after 3 years. What would you advise?’ For this case, LARCs would be an option: 55.9% would recommend hormonal IUD, 27.9% a cupper IUD and 16.2% an implant.

*Case 3*: ‘An 18-year old women would like to continue contraceptive use, but she often forgets to take the combination pill which she is currently using. What would you advise?’ Adherence to pill taking is problematic in this case, so one would expect a vast majority of respondents indicating methods that are less dependent on adherence. And indeed, injectables, vaginal ring and hormonal IUD score more than 50% here. It is however remarkable that almost 30% would recommend to continue the pill.

## Discussion

### Findings and interpretation

Based on these results, we can say that general practitioners do not routinely discuss LARCs with adolescents – let alone recommend them. This in itself is a worrying observation, because it indicates that adolescents are not always offered the necessary information to make an informed choice between a full range of modern contraception methods. This is contradictory to the recommendation of the World Health Organisation that ‘adolescents are eligible to use all the same methods of contraception as adults, and must have access to a variety of contraceptive choices’. (World Health Organization, 2016) This lack of informed choice may partly explain the low uptake of LARC among adolescents.

Another worrying finding is that most of the main reasons for hesitating to recommend LARCs to adolescents are provider-related barriers rather than reasons related to the well-being or comfort of the patients: the lack of confidence in own skills, the necessity to refer to a gynaecologist, and the fact that insertion and removal depend on an intervention by a medical doctor. This is in line with the finding that less than 40% of the respondents feels sufficiently skilled to place an implant and even fewer feel confident about placing an IUD. Lack of skills is clearly a barrier for prescribing an IUD to adolescents, but beyond that, there also seems to be an equipment problem (only half of the respondents indicate that their practice is sufficiently equipped to place implants and hormonal IUDs, and one in four to place copper IUDs) and also a remarkable lack of knowledge. About 30% describes his or her knowledge about implants as ‘limited’, and for copper IUD and hormonal IUD this is respectively 18% and 7%. The lack of knowledge is also illustrated by the fact that almost one third of the respondents does not seem to know that the pregnancy risk when using a copper IUD is in practice considerably lower than with a combination pill and that LARCs are cheaper on the long term.

In addition to lack of skills and knowledge – which could be assumed to equally apply to adult women than to adolescents – adolescents seem to face another barrier: prejudices and traditions that play a role in the reluctance of general practitioners to recommend LARCs to adolescents. The responses to the cases suggest that oral contraceptives remain the general script for contraceptive use among adolescents.

Trainings for general practitioners that target increasing knowledge and challenging attitudes are recommended.

### Strengths and weaknesses of the study

This study has a two main limitations. Firstly, the sample is potentially biased. As we used an online survey we have no control over the response bias and it is possible that more people with an interest in this topic have responded to the survey. However, based on overall data of general practitioners in Belgium, we are confident that the sample is a reflection of reality in term of socio-demographic characteristics. Secondly, the sample is small (79 respondents). Our assumption is that those who participated in the survey are already interested in the topic of adolescent contraceptive use and that therefore, the results may overestimate knowledge and positive attitudes towards and practices related to LARC use among adolescents. Therefore, we were mainly limited to descriptive statistics and were not able to demonstrate much significant differences between different groups of respondents. It is recommended that the study is repeated with a larger sample. Furthermore, it is likely that those responding to the online survey are more likely to be interested in the topic, and that the provider-related barriers are even larger than presented in this study.

Nevertheless, this study is among the first to shed light on the provider-related barriers to the use of LARCs among the vulnerable group of adolescents. By addressing several aspects related to LARCs, including knowledge, attitudes, skills and practices of general practitioners, we have gained insights in the barriers towards recommending and prescribing LARCs to adolescents on different levels.

### Differences in results and conclusion in relation to other studies

Few studies have assessed the knowledge, attitudes and practices of general practitioners in discussing and prescribing LARCs for adolescents.

Similar findings were reported in studies in Australia and Norway. In a study among 140 GPs in Norway (Bratlie, Aarvold et al. 2014) approximately 35% of GPs often discussed LARC methods when counselling but, due to a lack of implant insertion training, only a few frequently discussed implants during counselling. A research in Australia demonstrated that health care providers felt they lacked clinical experience to maintain competency inserting LARC.(Garrett et al. 2015) Furthermore, Bratlie et al (Bratlie et al. 2014) found oral contraceptives to remain the general script for contraceptive use among adolescents, while Kavanaugh et al. ( 2013) found several provider-related barriers to discussing and prescribing LARCs for adolescents including the extra time required to counsel young patients about LARCs and outdated clinic policies requiring multiple visits to obtain IUDs.

### Relevance of the findings

While teenage pregnancies in Flanders are rare, there are relatively high number of abortion among adolescents and young people. This indicates the suboptimal use of contraceptives in this group. While LARCs offer higher reliability in preventing pregnancies than SARCs, their use in this population group is low. Based on the data, we can say that (lack of) knowledge, skills and equipment of general practitioners constitute a barrier to uptake of LARCs by adolescents. There are undoubtedly several interactions and self-reinforcing mechanisms at work within the cluster skills-knowledge-prejudices-practices. Therefore it would be best to develop a comprehensive remediation approach addressing all components. Given the findings of this study and the important role that general practitioners can play in contraceptive counselling of adolescent girls, it is needed to address them with targeted information campaigns, and to pay proper attention to LARCs in the curricula of medical schools.

### Unanswered questions and future research

While a large number of adolescents go to the general practitioner to ask for contraceptive methods, a substantial number may also directly go to gynaecologists. Therefore, the knowledge, attitudes and practices of gynaecologists towards LARCs for adolescents should be studied to complement the results of this paper. Furthermore, it is important to get the perspective of the adolescents themselves towards LARCs. Insights into this triad - adolescents, general practitioners, gynaecologists - will provide the necessary understanding of barriers towards the use of LARCs in adolescents and suggestions for addressing these.
